# The Effect of Oseltamivir on the Disease Progression of Lethal Influenza A Virus Infection: Plasma Cytokine and miRNA Responses in a Mouse Model

**DOI:** 10.1155/2016/9296457

**Published:** 2016-03-08

**Authors:** Ashok K. Chockalingam, Salaheldin Hamed, David G. Goodwin, Barry A. Rosenzweig, Eric Pang, Michael T. Boyne II, Vikram Patel

**Affiliations:** ^1^DARS, OCP, OTS, CDER, US Food and Drug Administration, Silver Spring, MD 20993, USA; ^2^OCP, OTS, CDER, US Food and Drug Administration, Silver Spring, MD 20993, USA; ^3^DMD, OIR, CDRH, US Food and Drug Administration, Silver Spring, MD 20993, USA; ^4^OPQ, OLDP, CDER, US Food and Drug Administration, Silver Spring, MD 20993, USA; ^5^DPA, OTR, OPS, CDER, US Food and Drug Administration, Silver Spring, MD 20993, USA

## Abstract

Lethal influenza A virus infection leads to acute lung injury and possibly lethal complications. There has been a continuous effort to identify the possible predictors of disease severity. Unlike earlier studies, where biomarkers were analyzed on certain time points or days after infection, in this study biomarkers were evaluated over the entire course of infection. Circulating proinflammatory cytokines and/or miRNAs that track with the onset and progression of lethal A/Puerto Rico/8/34 (PR8) influenza A virus infection and their response to oseltamivir treatment were investigated up to 10 days after infection. Changes in plasma cytokines (IL-1*β*, IL-10, IL-12p70, IL-6, KC, TNF-*α*, and IFN-*γ*) and several candidate miRNAs were profiled. Among the cytokines analyzed, IL-6 and KC/GRO cytokines appeared to correlate with peak viral titer. Over the selected 48 miRNAs profiled, certain miRNAs were up- or downregulated in a manner that was dependent on the oseltamivir treatment and disease severity. Our findings suggest that IL-6 and KC/GRO cytokines can be a potential disease severity biomarker and/or marker for the progression/remission of infection. Further studies to explore other cytokines, miRNAs, and lung injury proteins in serum with different subtypes of influenza A viruses with varying disease severity may provide new insight into other unique biomarkers.

## 1. Introduction

Influenza viruses are a major cause of acute viral infections of the respiratory tract. The severity of influenza infection can range from asymptomatic infection to primary viral pneumonia and death. Seasonal influenza A viruses infect 5–15% of the global population and results in more than 500,000 deaths annually [1]. In addition to seasonal epidemics, occasionally the emergence of novel influenza strains can result in widespread pandemics and extensive morbidity and mortality. The 1918 H1N1 pandemic influenza virus killed an estimated 50 million people and the recent 2009 influenza A(H1N1)pdm09 virus caused more than 284,000 deaths globally during the first 12 months of virus circulation due to enhanced virulence compared to seasonal influenza viruses [2].

Antiviral drugs are essential means of managing severe influenza infection to reduce the duration of symptoms and improve clinical outcomes. Antiviral drugs like adamantane derivatives (amantadine and rimantadine) and neuraminidase inhibitors (oseltamivir and zanamivir) are used for treating influenza infection. Among the antivirals, oseltamivir has been used as a drug of choice for influenza infection. It is stockpiled in many countries as a medical counter measure to possible pandemics. It has been shown that oseltamivir reduces cellular and cytokine inflammatory response in the lung and thereby abrogates the immunopathology when given before infection or after infection in a mouse model of nonlethal influenza infection [3]. But, in case of lethal influenza infection, higher doses and prolonged oseltamivir therapy are needed early to block virus replication and prevent triggering dysregulation of immune response. Once triggered, the immune mediated tissue damage is possibly not very sensitive to the presence of antiviral agents [4–6].

Influenza virus infection in immunocompromised, elderly, or chronically ill populations leads to acute lung injury and possibly lethal complications. A combination of viral virulence and uncontrolled inflammatory responses to the virus has broadly been suggested to be a predominant mechanism for severe lung pathology [7]. There has been a continuous effort to identify the possible predictors of the severity of influenza virus infection in humans [8, 9]. The ideal predictors would be those that are readily obtainable from the host and easily performed in the laboratory, guide therapy and predict the beneficial or harmful aspects of host defense following their initial diagnosis. Translational biomarkers that bridge between animal and human trials would be invaluable in predicting the efficacy of current or new medical countermeasure agents and help determine the optimal dosing paradigms depending on the stage of infection [10]. Development of plasma (circulating) biomarkers that can identify one or more features of different phases of influenza virus infection in animal model will facilitate the early diagnosis and the course of treatment for fatal infections in humans.

Inflammation due to viral diseases is rapid and nonspecific host defense mechanism against infection is intricately regulated by a network of mediators which include inflammatory cytokines. The extent of lung injury in severe influenza infections may be in part due to overly exuberant or dysregulated innate inflammatory responses [11]. Several studies have examined correlation of flu disease parameters such as viral shedding and symptom scores with levels of IL-6 [12, 13], which was recently reported as a potential biomarker of severity in pandemic influenza infections [14]. However, there was no study in the literature to find the correlation of this cytokine over the course of influenza A virus infection and its effect on treatment. Apart from inflammatory mediators, circulating microRNAs (miRNAs) are increasingly the target of biomarker development because of their high stability in body fluids, making them ideal and powerful informative markers for cerebrovascular [15] and cardiovascular disorders [16] and certain cancer conditions [17]. Similarly, circulating miRNAs also are applied for potent detection of infectious diseases including influenza A viruses [18–21].

The above described studies were conducted to identify a biomarker on particular time point or days after infection, but not over a course of infection. The time course study of influenza A virus infection will provide a correlation between the biomarker and the disease severity and/or treatment prognosis for the established antivirals as well as aid in the development of new medical countermeasures. Thus, the goal of this current study was to identify disease specific circulating inflammatory and/or miRNA markers that track with the onset and progression of lethal influenza A virus infection and their response to prophylactic and therapeutic oseltamivir (Tamiflu) treatment in a Balb/c mouse model.

## 2. Materials and Methods

Mouse adapted influenza A virus, A/Puerto Rico/8/34 (H1N1) (PR8) was a generous gift from Dr. Daniel R. Perez, University of Maryland. Virus stocks were prepared by inoculating in 10-day-old embryonated chicken eggs and titrated in Madin-Darby Canine Kidney (MDCK) cells. Virus stocks were stored at −80°C prior to use. Oseltamivir phosphate (OP) purchased from Toronto Research Chemicals, Canada, was used for the study. Female Balb/c mice (8–10 weeks of age) were purchased from Taconic Farms Inc. (Hudson, NY, USA) and used in influenza studies. Mice were maintained in clean microisolator cages and controlled temperature and humidity with a 12-hour light and dark cycle. Mice were given standard animal feed and water* ad libitum.*

### 2.1. Virus Infection and Oseltamivir Dosing

All mouse procedures were performed in accordance with Center for Drug Evaluation and Research (CDER) Animal Care and Use Committee guidelines. All animal procedures were performed in a certified class II biosafety cabinet. In each study, forty female Balb/c mice were randomized into 10 groups of 4 animals per group. Mice were anesthetized with 2.5% isoflurane and infected intranasally with 100 or 1000 times the 50% tissue culture infective dose (TCID50) for therapeutic groups and 1000 TCID50 for prophylactic groups in a volume of 50 *µ*L. Throughout the animal experiments, animal temperature, weights, and survival were recorded daily over a period of 10 days after infection.

Oseltamivir phosphate was prepared in sterile water and administered at 10 mg/kg/day by oral gavage twice daily for 5 days, with an 8-hour interval between doses. The prophylactic group was dosed 2 hours prior to infection and the therapeutic group was dosed 24 hours after infection. Infected control mice groups were oral gavaged with sterile water according to the oseltamivir phosphate treatment schedule.

### 2.2. Lung Viral Load Quantitation

On days 1 through 10 after infection, mice (*n* = 4) were euthanized, lungs were collected, homogenized in phosphate buffered saline (supplemented with 100 U/mL penicillin, 100 *µ*g/mL streptomycin, and 0.25 *µ*g/mL of amphotericin B), and centrifuged, and the supernatants were used to infect MDCK cells in quadruplicate. Cells were incubated for 72 hours at 37°C and 5% CO_2_. After incubation, cell culture supernatants were tested for the presence of virus by hemagglutination assay using 0.5% (v/v) chicken red blood cells (Innovative Research, MI, USA). Viral loads were determined as the reciprocal of the dilution at which 50% of the wells positive for viral infection were expressed as TCID50 per mL in logarithmic scale.

### 2.3. Plasma Samples

On days 1 through 10 after infection, blood samples were collected (*n* = 4) intracardially from anesthetized mice in Minicollect with EDTA tubes (Greiner Bio-one GmBH, Austria). Plasma was separated from blood samples by centrifuging 13000 rpm for 10 min. at 4°C and the supernatant was recentrifuged at 14,000 ×g, at 4°C, for 10 min. 55 *µ*L of plasma sample was aliquoted for miRNA analysis. The remaining plasma was mixed with the protease inhibitor cocktail (cOmplete ULTRA, MINI EDTA-free, Roche) for cytokine quantitation. All plasma samples were stored at −80°C until analysis.

### 2.4. Cytokine Quantitation and miRNA Profiling

Plasma concentrations of multiple cytokine and chemokines were obtained using a mouse proinflammatory 7-plex (IL-1*β*, IL-12p70, IFN-*γ*, IL-6, KC/GRO, IL-10, and TNF-*α*) ultrasensitive kit (Meso Scale Diagnostics, MD, USA). The analytes limit of detection (LOD), lower limit of quantification (LLOQ), and upper limit of quantification (ULOQ) were determined before the study samples were analyzed. ELISAs were run essentially according to the manufacturer's instructions and were read on the MSD 96-well plate reader. The concentrations in replication were determined by standard curve method. The data were expressed as means ± SD. Student's two tailed *t*-test *p* values ≤ 0.05 were considered statistically significant.

Total RNA (primarily miRNA and small RNA) was isolated from plasma samples using miRNeasy serum/plasma kit (Qiagen, USA) and QIAcube automated robotic workstation according to the manufacturer's protocol. Total RNA was eluted in 15 *µ*L of RNase-free water and stored at −80°C until analysis. Reverse transcription reactions were performed in a volume of 7.5 *µ*L containing 3 *µ*L of purified RNAs using 1x Megaplex RT primers (Rodent pools A and B v3.0, Life Technologies) and the resulting cDNA was preamplified using Megaplex PreAmp primers (Life Technologies) before the qPCR reaction. Preamplified cDNA was diluted with 75 *µ*L of 0.1x TE and stored at −20°C. Quantitative PCR assays were performed using the TaqMan Array Rodent miRNA A + B card set v3.0 kit (Life Technologies). Each card contains a total of 384 TaqMan miRNA assays, able to quantify 750 rodent miRNAs. Every card contains three endogenous controls and one negative control. Qualitative PCR was performed in ViiA 7 Real-Time PCR system (Life Technologies) according to the manufacturer's protocol. The data were collected and processed using the ViiA 7 and SDS v2.3 software.

miRNA expression levels were evaluated using comparative cycle threshold (Ct) method. Ct values ranged from 0 to 40. Out of 750 miRNAs analyzed from the TaqMan miRNA assays, 48 miRNA of interest were selected for further analysis using custom developed TaqMan miRNA plates. Of these 48 miRNAs, 44 miRNAs were selected based on Ct values <35-fold and ≥1.5-fold difference for a specific miRNA between the PBS control and virus treatment groups in one or more time points. Three miRNAs (miR-9^*∗*^, miR-1935, and miR-741), which were not amplified, were included as negative control for analysis. The miRNA U6 snRNA was included as a plate control. All the procedures for the 48 miRNA amplification were the same as the TaqMan Array cards except the RT reaction volume that was 12 *µ*L, the preamplified cDNA was diluted with 175 *µ*L of 0.1x TE, and 1 *µ*L of the PreAmp product was used in the qPCR reaction.

Relative expression levels were calculated using the 2^−ΔΔCt^ method. First, miRNA cycle threshold (Ct) values were normalized to U6 snRNA (as this miRNA did not show much variation across the plates) and then to the average control (virus infected without oseltamivir treatment) value. Significant differences between oseltamivir treated and untreated groups were identified using two tailed student's *t*-test. A *p* value ≤0.05 was considered statistically significant.

## 3. Results

### 3.1. Effect of Oseltamivir on Influenza Virus Disease Progression

Balb/c mice infected with mouse adapted PR8 (H1N1) virus caused lethal disease over the course of infection as evidenced by weight loss and death. Both disease symptoms and severity appeared to be delayed in the prophylactic treatment group. However, no significant differences in these endpoints were observed when therapeutic treatment and control groups were compared.

**Figure 1:**
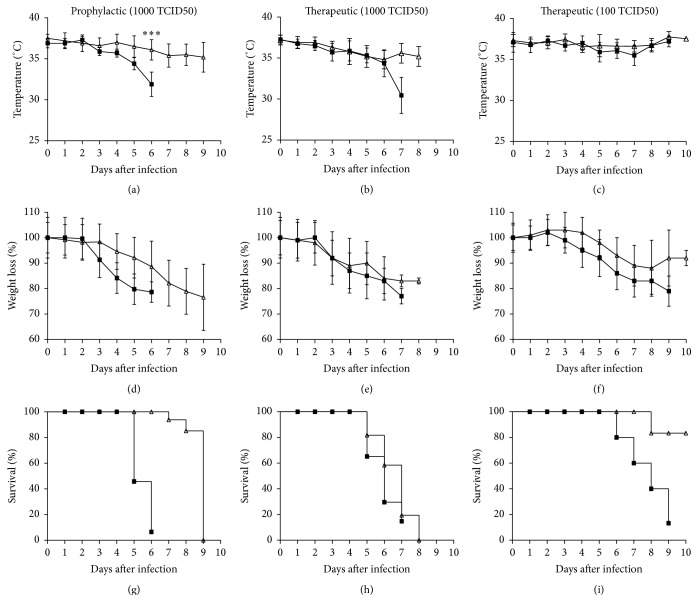
Prophylactic oseltamivir treatment delay severity of lethal influenza infection progression in mice. Body temperature ((a), (b), and (c)), weight loss ((d), (e), and (f)), and survival ((g), (h), and (i)) of Balb/c mice infected with mouse adapted A/Puerto Rico/8/34 (H1N1) virus and treated with oseltamivir phosphate. For the prophylactic group ((a), (d), and (g)), groups of 40 mice were infected with 1000 TCID50 virus and treated 2 h before infection with oseltamivir. The therapeutic groups were infected with 1000 TCID50 ((b), (e), and (h)) or 100 TCID50 ((c), (f), and (i)) virus and treated 24 h after infection. Prophylactic and therapeutic groups (Δ) were administered with oseltamivir 10 mg/kg by oral gavage twice daily for 5 days. An infected group (■) gavaged with distilled water was added as control for each experiment. To ascertain significance, two tailed and two sample unequal variances Student's *t*-test was used (^*∗∗∗*^ *p* < 0.001). The Kaplan-Meier method was used to estimate the probability of survival.

As shown in Figures 1(g)–1(i), intranasal inoculation of 1000 TCID50 of A/PR/8/34 (H1N1) virus in untreated control mice resulted in a 100% mortality rate on days 6 to 7 after infection and, for 100 TCID50 dose group, 100% mortality was observed on day 9 after infection. Prophylactic treatment with oseltamivir significantly delayed mortality by 3 days (Figure 1(g)). On the other hand, therapeutic treatment with oseltamivir significantly delayed mortality by 1 day for the 100 TCID50 virus dose group (Figures 1(h) and 1(i)). A sudden drop in mean body temperature (~2–6°C) was observed on and after day 4 after infection for the untreated group infected with 1000 TCID50 of PR8 virus. The 1000 TCID50 PR8 virus infected groups treated either prophylactically or therapeutically with oseltamivir showed a gradual loss in mean body temperature (~2–3°C) until humane endpoints were met (Figures 1(a) and 1(b)). No significant difference in body temperature was observed for the mice infected with 100 TCID50 of PR8 virus either untreated or treated therapeutically with oseltamivir (Figure 1(c)). On day 6 or 7 after infection, the 1000 TCID50 virus alone infected group lost more than 20% body weight. The prophylactic treatment group showed a gradual loss in body weight compared to therapeutic treatment group, in which the weight loss was sudden after 2 days after infection and mice in both groups lost more than 20% body weight on days 9 and 8 after infection, respectively (Figures 1(d) and 1(e)). Body weight loss for the 100 TCID50 of PR8 virus infected group was observed on day 3 and lost more than 20% on day 7 after infection, whereas the oseltamivir treated group at this dose level started losing body weight on day 4 until day 8 and later it regained weight slowly (Figure 1(f)).

**Figure 2:**
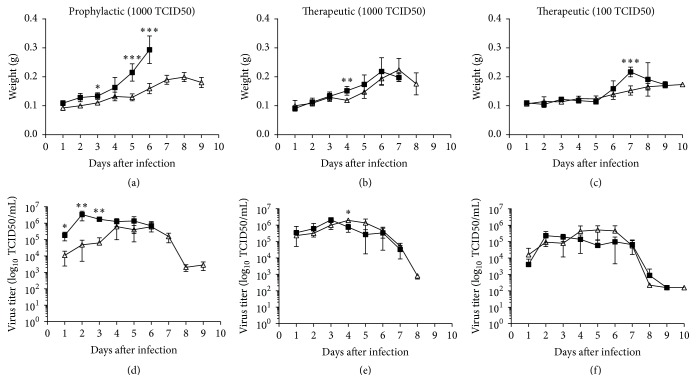
Prophylactic oseltamivir treatment decreases lung viral titer on early days of lethal influenza infection in mice. Lung weight ((a), (b), and (c)) and viral titers ((d), (e), and (f)) of Balb/c mice infected with mouse adapted A/Puerto Rico/8/34 (H1N1) virus and treated with oseltamivir phosphate. For the prophylactic group ((a) and (d)), groups of 40 mice were infected with 1000 TCID50 virus and treated 2 h before infection with oseltamivir. The therapeutic groups were infected with 1000 TCID50 ((b) and (e)) or 100 TCID50 ((c) and (f)) virus and treated 24 h after infection. Prophylactic and therapeutic groups (Δ) were administered with oseltamivir 10 mg/kg by oral gavage twice daily for 5 days. An infected group (■) gavaged with distilled water was added as control for each experiment. On days 1–10 after infection, mice were sacrificed and viral titers in lung homogenates were determined by endpoint titration in MDCK cells. To ascertain significance, two tailed, two sample unequal variances Student's *t*-test was used (^*∗*^ *p* < 0.05, ^*∗∗*^ *p* < 0.01, and ^*∗∗∗*^ *p* < 0.001).

Lung weight and viral titer were also analyzed to evaluate the efficacy of oseltamivir on viral pathogenicity in mice. The humane endpoint was determined at 20% weight loss. On days 1–10 after infection, four mice per group were sacrificed to determine lung weight and viral titer. The lungs of infected mice were enlarged and edematous due to fluid accumulation. As shown in Figures 2(a)–2(c), the weight of the lungs increased gradually from 2 to 10 days after infection. Significant differences in the mean lung weight were observed between prophylactic treatment group and untreated control group on days 5 and 6 after infection (*p* < 0.001) (Figure 2(a)). This difference in lung weight was not present in the therapeutic group except on day 4 after infection (*p* ≤ 0.01) (Figure 2(b)), and, at 100 TCID50 virus dose level, this difference in lung weight was observed only on day 7 after infection (*p* ≤ 0.001) (Figure 2(c)). Viral titers from the infected and oseltamivir treated lung tissues were estimated over the course of infection as a disease progression indicator. At 1–3 days after infection, virus titers in the prophylactic treatment group were significantly lower than the untreated group (*p* < 0.01), but at later days the viral loads were similar (Figure 2(d)). As shown in Figures 2(e) and 2(f), no significant differences in the kinetics of viral load were observed throughout the course of infection between the therapeutic and untreated groups at both infection dose levels except on day 4 after infection, where *p* value of <0.05 was observed for the 1000 TCID50 virus infected therapeutic group.

### 3.2. Relationship of Cytokines with Disease Progression

**Figure 3:**
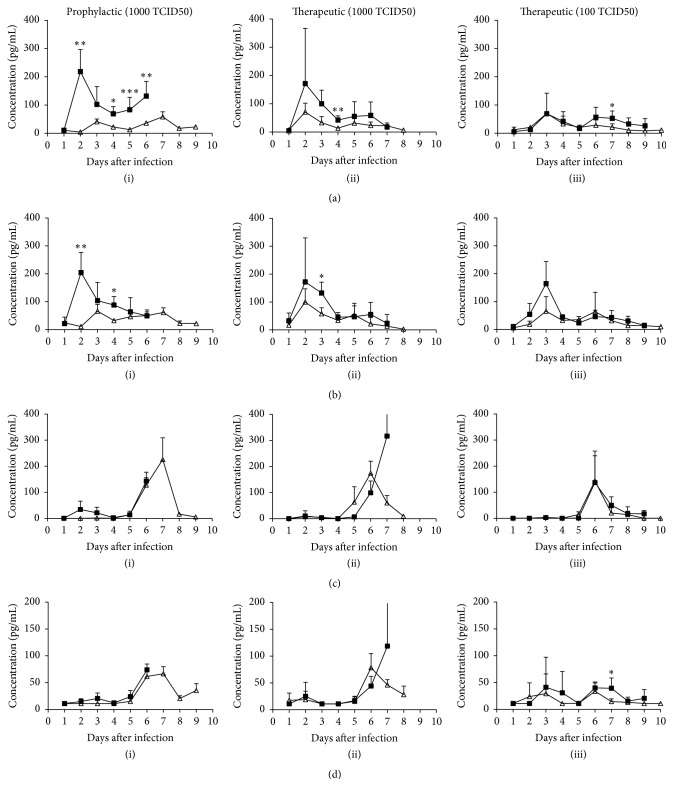
Effect of oseltamivir treatment on cytokine levels in lethal influenza A virus infected mice. Changes in cytokine concentrations [IL-6 (a), KC/GRO (b), IFN-*γ* (c), and IL-10 (d)] over time in plasma samples from mice infected with lethal mouse adapted influenza A/Puerto Rico/8/34 (H1N1) virus and treated either with oseltamivir phosphate or water. For the prophylactic group [(a)–(d) (i)], mice were infected with 1000 TCID50 virus and treated 2 h before infection with oseltamivir. The therapeutic groups were infected with 1000 TCID50 [(a)–(d) (ii)] or 100 TCID50 [(a)–(d) (iii)] virus and treated 24 h after infection. Prophylactic and therapeutic groups (Δ) were administered with oseltamivir 10 mg/kg by oral gavage twice daily for 5 days. An infected group (■) gavaged with distilled water was added as control for each experiment. On days 1–10 after infection plasma samples were collected from sacrificed mice and cytokine levels were measured by Mesoscale ELISA. Levels were indicated as pg/mL of plasma. For the purpose of analysis, cytokine levels below the detection limit were set to the lower limit of detection in each case. To ascertain significance, two tailed, two sample unequal variances Student's *t*-test was used (^*∗*^ *p* < 0.05, ^*∗∗*^ *p* < 0.01, and ^*∗∗∗*^ *p* < 0.001).

The serum concentrations of seven proinflammatory cytokines (IL-1*β*, IL-10, IL-12p70, IL-6, KC-GRO, TNF-*α*, and IFN-*γ*) were measured and compared between the different treatment groups and their correlation with disease progression. Substantial increases in IL-6 cytokine levels were observed at 2 days after infection and gradually decreased over the course of infection in 1000 TCID50 infected and untreated control groups (Figures 3(a)(i) and 3(a)(ii)). In the prophylactic 1000 TCID50 group, IL-6 cytokine levels were significantly reduced (*p* < 0.05, 0.01, 0.001) compared to the untreated control group at 2 days after infection and at later days except on day 3 (Figure 3(a)(i)). The therapeutic 1000 TCID50 treatment group showed reduced expression levels of these cytokines, but the differences were not significant statistically from untreated control group throughout the course of infection except on day 4 (*p* < 0.01) (Figure 3(a)(ii)). In the case of the therapeutic 100 TCID50 treatment group, IL-6 levels peaked at day 3 after infection and no significant difference in the expression levels of these cytokines from the untreated control group was observed until day 5 after infection, and, on later days, the drug treated group showed reduced expression with significant differences on day 7 after infection (*p* < 0.05) (Figure 3(a)(iii)).

The chemokine KC/GRO peaked at 2 days after infection for 1000 TCID50 group and at 3 days after infection for 100 TCID50 control group (Figures 3(b)(i)–3(b)(iii)). prophylactic group and 1000 TCID50 therapeutic group showed significant difference in the KC/GRO expression level on days 2 (*p* < 0.01) and 4 (*p* < 0.05) and day 3 (*p* < 0.05) after infection, respectively, compared to untreated control group (Figures 3(b)(i) and 3(b)(ii)). No difference in the expression of KC/GRO was observed between oseltamivir treated and untreated mice in the 100 TCID50 group (Figure 3(b)(iii)). The expression of IL-6 and KC cytokines appeared to track with disease progression, nonsequentially with viral load, but the differences among oseltamivir treated and untreated groups were not significant at these infection dose levels except for the IL-6 cytokine in the prophylactic group. The cytokine IFN-*γ* levels were undetected at early days of infection, but the levels increased after 5 days after infection with peak levels on 6 or 7 days after infection for therapeutic and prophylactic groups, respectively. The expression levels of IFN-*γ* were not statistically significant among oseltamivir treated and untreated groups (Figures 3(c)(i)–3(c)(iii)). The cytokine IL-10 expression level follows the similar trend with IFN-*γ* for the prophylactic and therapeutic 1000 TCID50 groups (Figures 3(d)(i) and 3(d)(ii)). The therapeutic 100 TCID50 group did not show any particular trend in the IL-10 expression for the entire study period except on day 7 after infection (*p* ≤ 0.05) (Figure 3(d)(iii)).

The expression levels of IL-1*β*, IL-12p70, and TNF-*α* were not detected above the detection limit for most of the study time points for the prophylactic and 1000 TCID50 therapeutic groups and untreated control groups (data not shown). The therapeutic 100 TCID50 group showed transient increase in the expression levels of TNF-*α* and IL-1*β* throughout the course of infection and of the IL-12p70 on days 3 and 4 after infection. However, no differences in the expression of these cytokines were observed among oseltamivir treated and untreated groups (ESM_1.docx in Supplementary Material available online at http://dx.doi.org/10.1155/2016/9296457).

### 3.3. Global miRNA Expression Analysis and Disease Progression

To investigate the effect of influenza virus infection on host miRNAs, we profiled plasma miRNAs via the TaqMan Low Density Array Rodent MiRNA Panel A + B, which could simultaneously detect the expression levels of 750 miRNAs. The analysis was limited to miRNAs that were expressed in all the analyzed days from the virus infected groups. Upon PR8 virus infection, 368 miRNAs were found to be expressed in pooled plasma (4 mice per day) from 100 TCID50 infected groups on 1, 3, 7, and 9 days after infection and 1000 TCID50 infected groups on 1, 3, 5, and 7 days after infection, whereas 447 miRNAs were detected in plasma from healthy controls (ESM_2.xlsx).

**Figure 4:**
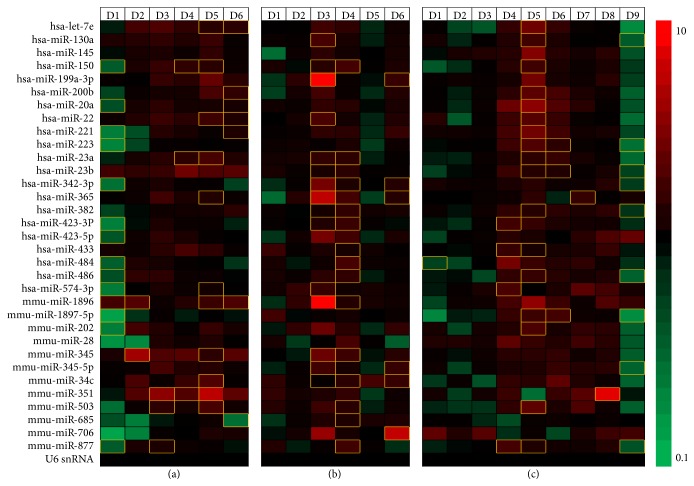
Analysis of circulating miRNA profiles in oseltamivir treated/untreated mice infected with lethal influenza A virus. Heat map of prophylactic [(1000 TCID50) (a)] and therapeutic [(1000 TCID50) (b) and (100 TCID50) (c)] effect of oseltamivir phosphate on the plasma miRNA levels of A/Puerto Rico/8/34 (H1N1) virus infected mice. miRNAs were analyzed from the plasma of virus infected and oseltamivir treated as well as virus alone infected mice from days 1 to 9 after infection. The various shades of red represent miRNAs that were upregulated and the various shades of green represent miRNAs that were downregulated in prophylactic and therapeutic groups, when compared with the virus alone infected group. Statistically significant unique miRNA expression patterns (*p* ≤ 0.05 and 0.01) from days 1 to 9 after infection were depicted as yellow borders. The heat map depicts the ratio of group averages for drug treated/virus infected alone and the scale of the heat map is 0.1–10.

Among the 48 miRNAs analyzed further, nine miRNAs did not amplify in one or more time point after infection, along with the three negative control miRNAs included in the assay. As our experiment did not include miRNAs from time matched healthy control, we compared the miRNAs expressed in the oseltamivir treated group with the virus alone infected group (ESM_3.xlsx, ESM_4.xlsx and ESM_5.xlsx). As seen in Figure 4, most of the selected miRNAs were initially downregulated depending on oseltamivir treatment regimen and once the disease severity progresses, the upregulated miRNA increased compared to virus alone infected groups at all days after infection. In the prophylactic treatment group, more miRNAs were downregulated on day 1 after infection than in the therapeutic groups. In the days following infection, the numbers of upregulated miRNAs increased and downregulated miRNAs decreased. Similar transient changes in the plasma miRNA levels were also observed for the therapeutic treatment groups (Figure 4). These patterns of miRNA expression indicate that prophylactic oseltamivir treatment delayed the upregulation of plasma miRNA levels by virus infection. The higher expression levels of plasma miRNAs observed at days 3 and 4 with 1000 TCID50 and at day 5 with 100 TCID50 compared to virus alone controls correlate with virus titer. This data suggests possible correlation of circulating miRNA levels with the virus titer, severity of infection, and mortality of the animals in the prophylactic and therapeutic treatment groups.

Next, we examined the miRNAs from the oseltamivir treated groups with the untreated groups to determine whether oseltamivir treatment leads to changes in the miRNA expression profile.  Table 1 demonstrates the number of significantly (*p* ≤ 0.05) upregulated and downregulated miRNAs overtime after infection due to oseltamivir treatment compared with untreated group. Of the analyzed miRNAs, miR-150, miR-20a, miR-22, miR-23a, miR-221, miR-223, miR-342-3p, miR-423-3P, miR-484, miR-486, and miR-877 were commonly found in all the treatment groups, but at different days after infection.

## 4. Discussion

Biomarkers are measurable characteristics and evaluated as an indicator of normal biologic processes, pathogenic processes, or pharmacologic responses to a therapeutic intervention. The biomarker identification for influenza A virus infection is important in two different ways; namely, when obtained early in the course of infection, it could predict the prognosis of disease and it is useful for the development of a new therapy as there continues to be a concern about the effectiveness of neuraminidase inhibitors and the emergence of resistant strains. Several studies in animal models and humans have attempted to identify inflammatory or circulatory sera/plasma miRNA markers and their correlation with disease severity to aid in early prognosis and thereby its treatment options [3, 8, 10, 14, 20, 21]. Unlike earlier studies, where biomarkers were analyzed on particular days after infection, in this study we conducted a systematic lethal PR8 virus infection progression in a mouse model, tracking multiple inflammatory cytokines and miRNAs over a course of 10 days to identify the correlation of disease severity with inflammatory and miRNA marker profiles and how these markers alter in response to oseltamivir treatment.

Oseltamivir administration has been shown to be effective in the treatment and prevention of influenza infection [22, 23], but the dose and duration of oseltamivir treatment depend on the viral inoculum and its virulence [5, 24]. We observed significant reduction in lung viral titers on initial days of infection and thereby delayed mortality in the prophylactic group. There was no difference in lung viral titers between therapeutic groups and virus alone infected group. The virus titers peaked at 4 days after infection for both the prophylactic and the therapeutic groups compared to 2 days after infection for virus alone infected group. Apart from the delay in the peak viral titers, the therapeutic group showed an increase in viral titers on 5 and 6 days after infection compared to virus alone infected group which implies that oseltamivir is able to inhibit viral release and thereby replication during the initial infection period, but when the antiviral effect subsides, residual viral infection in the infected lung may lead to a rise in viral titer. No differences in the lung viral titer were reported for the mice infected with H3N1 (Mem71) strain of sublethal influenza A virus and viral titers measured on 1 and 2 days after infection for therapeutic group and 3 and 5 days after infection for prophylactic group [3]. This reinforces the point that an excessive immune response is likely to initiate tissue damage during lethal influenza virus infection that will not be improved by antivirals that target viral replication. But extending oseltamivir treatment and higher doses while lung virus titers remain high had shown better antiviral effect and thereby prolonged survival [6].

Clinical studies in humans with acute influenza, small animal model testing, and* in vitro* laboratory experiments have revealed a number of inflammatory and immune mediators that appear to play a significant role in the initial host response to influenza virus infection [8, 14, 25, 26]. In our study, IL-6 levels correlated with viral titer throughout the course of infection and prophylactic treatment significantly reduced the IL-6 levels. Similarly, therapeutic administration reduced IL-6 levels, but the level of reduction did not reach statistical significance except on certain days after infection. The chemokine KC/GRO levels were increased on early infection and gradually declined at later days. Oseltamivir administration reduced the expression of KC/GRO levels during the initial acute febrile phase of infection. This observation strongly supports the relationship between IL-6 and KC production with peak viral titer and also early or prophylactic oseltamivir treatment abrogated IL-6 response and its effect on the host.

We did not find significant associations between viral titer, body weight loss, and levels of IFN-*γ*, IL-10, TNF-*α* and IL-1*β*. TNF-*α* and IL-1*β* are important proinflammatory cytokines and contribute directly to the severity of gross and histologic lung lesions in the influenza infected mice. We found that TNF-*α* and IL-1*β* levels were below the detection limit for most of the days after infection (data not shown) for both high dose (1000 TCID50) groups treated with oseltamivir either prophylactic or therapeutic regimen. The results are in accordance with the study by [11], where 5 × 10^5^ plaque forming units of PR8 virus infection only transiently increased the TNF-*α* level at 2 days after infection. Also it has been shown that viral strain differences (BJx109 and PR8 virus) in the local inflammatory response in stimulating the release of type I IFNs [27]. The IFN-*γ* and IL-10 expression level significantly increased at day 6 after infection, later both cytokine levels were decreased to below the detection limit and oseltamivir treatment did not affect the expression levels of these cytokines. This finding is in agreement with the earlier study, where fatal PR8 virus infection increased the level of IFN-*γ* and IL-10 at day 6 after infection, but it rapidly decreased to control level later on [28]. We conducted a separate experiment, in which the sera collected from control as well as oseltamivir alone treated animals did not show any difference between the groups for the analyzed cytokines and at least for five miRNAs (miR-22, miR-206, miR-494, miR-1897-5p, and U6 snRNA) (data not shown). Thus, we did not include a time matched control set in our other experiments.

miRNAs in body fluids such as plasma, serum, urine, and saliva have been investigated widely in patient samples and animal models and have been revealed as potential biomarkers of various diseases [15–17]. Tambyah et al. showed changes occurring in the miRNA transcriptome of patient's blood samples, where 75 miRNAs were significantly upregulated while 118 were downregulated from blood samples of H1N1 infected patients prescribed with Tamiflu compared to healthy controls [20]. In another study, the following miRNAs (miR-150, miR-31, miR-155, miR-29a, miR-29b, miR-342-5p, and miR-886-3p) were highly dysregulated in PBMCs from critically ill patients infected with pandemic H1N1 influenza virus compared to healthy controls [29]. Most recently, evaluation of serum miRNA profile from H7N9 avian influenza virus infected patients showed significant elevation in the levels of miR-17, miR-20a, miR-106a, and miR-376c compared with healthy individuals [21]. In accordance with the above studies, we also observed that the following miRNAs were found to be significantly dysregulated (let-7e, miR-20a, miR-22, miR-23a, miR-145, miR-150, miR-200b, miR-221^*∗*^, miR-223, miR-342-3p, miR-345, miR-423-3p, miR-423-5p, miR-484, and miR-574-3p) in mice infected with PR8 (H1N1) virus and treated with Tamiflu compared to virus alone infected group. But there were differences in the regulation (up- or downregulation) of these miRNAs at different stages of infection.

Analyses of miRNAs from influenza A virus infected mouse lungs were found to be altered similar to body fluids. The upregulated plasma miRNAs, miR-145, miR-223, miR-1897-5p, miR-706, and miR-877 observed in our study were also found to be upregulated, except for miR-145 on day 5 after infection, in the lungs of mice infected with 10,000 TCID50 of PR8 virus [30]. Upregulation of miRNA and its associated disease pathology were also observed in mice infected with A/Vietnam/1203/04, A/California/04/09, and A/Texas/36/91 strains. More miRNAs were upregulated in mice infected with highly pathogenic avian influenza strain A/Vietnam/1203/04 or pandemic strain A/California/04/09, whereas more miRNAs were downregulated in mice infected with nonlethal seasonal strain A/Texas/36/91 [31]. In our study, upregulation of the miRNAs in the mice infected with PR8 virus alone and gradual upregulation of miRNAs over the course of infection in oseltamivir treated groups correlated with disease severity. Several other studies further demonstrated an up- or downregulation of miRNAs in the lungs from mice infected with various influenza viruses [30, 32, 33]. Apart from miRNA dysregulation observed from the authors of tghese studies with various influenza viruses, specific miRNAs were shown to upregulate and contribute to virulence. The upregulation of miR-200a and miR-223 was shown to contribute to virulence of the r1918 influenza virus [32], highly pathogenic avian influenza virus A/Vietnam/04/09, and pandemic A/California/04/09 [31] and the upregulation of miR-223 for pandemic H1N1 and PR8 virus [30]. We observed that miR-223 was downregulated in the prophylactic group and upregulated in the therapeutic group in the present study which may also be involved in the difference in disease severity.

The current study represents the systematic investigation of the lethal influenza A virus disease progression in a mouse model and its responses with inflammatory cytokines and plasma miRNAs and how these markers change in response to oseltamivir treatment. Severe PR8 (H1N1) virus infection induced viral dose dependent elevation of IL-6 and KC/GRO level in early phase of infection, which corresponded to peak viral titer and the course of infection, but only IL-6 level showed significant reduction after prophylactic oseltamivir treatment. As found in other studies, our studies further confirm that IL-6 may serve as an important marker for severe complications following influenza infection. It is difficult to determine the usefulness of other cytokines, where IFN-*γ* and IL-10 were elevated late in the infection and the TNF-*α*, IL-1*β*, and IL-12p70 cytokines were randomly elevated and did not respond to oseltamivir. Upon analysis of selected miRNAs, we also found that significant differences in the expression profile of certain miRNAs (let-7e, miR-20a, miR-22, miR-23a, miR-145, miR-150, miR-200b, miR-221^*∗*^, miR-223, miR-342-3p, miRNA-345, miR-423-3p, miR-423-5p, miR-484, and miR-574-3p) in response to oseltamivir treatment compared to untreated group. While many groups have demonstrated the presence of miRNA changes using* in vitro* and* in vivo* (lung tissue or plasma) systems on certain days after infection with different influenza A virus subtypes, very few miRNAs are commonly dysregulated among the studies. Unlike other studies where miRNAs were analyzed on a particular time point or days after infection, our study over a period of disease course reveals that different miRNAs were up- or downregulated on different days after infection with varying severity of infection and treatment regimen. Also, it is difficult to predict whether the proinflammatory cytokines and miRNA data observed with PR8 (H1N1) virus infection in this study as well as the results from earlier studies with different influenza A virus strains could be applied universally to all influenza A virus infections. The variation in the expression of specific miRNA was also reported for other important viral infections of humans like hepatitis B virus, hepatitis C virus, human papilloma virus, and human immunodeficiency virus-1 infection between clinical strains of the same virus, different tissues/fluids analyzed, and different research groups [34]. Therefore, further studies with different subtypes of influenza A viruses with varying severity over a course of infection will provide a new insight into unique biomarker regulation.

In conclusion, these results suggest that IL-6 and KC/GRO cytokines can be a potential disease severity biomarker and/or marker for the progression/remission of influenza A virus infection. Further studies to explore other cytokines, miRNAs, and lung injury proteins in serum might offer novel unique targets to predict the course of different subtypes of influenza A virus infection and thereby effective therapeutic intervention against severe influenza A virus infection.

## Supplementary Material

ESM_1.docx Effect of therapeutic oseltamivir treatment on cytokine levels in lethal influenza A virus infected mice. Changes in cytokine concentrations [TNF-α (a), IL-1β (b), and IL-12p70 (c)] over time in plasma samples of mice infected with 100 TCID50 lethal mouse adapted influenza A/Puerto Rico/8/34 (H1N1) virus and treated therapeutically either with oseltamivir phosphate or water. Therapeutic groups (Δ) were administered 24 h post-infection with oseltamivir 10 mg/kg by oral gavage twice daily for 5 days. An infected group (■) gavaged with distilled water was added as control for each experiment. On day 1 – 10 post-infection, plasma samples were collected from sacrificed mice and cytokine levels were measured by Mesoscale ELISA. Levels were indicated as pg/ml of plasma. For the purpose of analysis, cytokine levels below the detection limit were set to the lower limit of detection in each case. (DOCX 164 kb).
ESM_2.xlsx Global rodent miRNA expression profile of healthy and A/Puerto Rico/8/34(H1N1) (PR8) virus (100 and 1000 TCID50) infected Balb/c mouse plasma samples. (XLSX 135 kb).
ESM_3.xlsx Selected candidate miRNA expression profile comparison of Balb/c mice infected with 1000 TCID50 of mouse adapted A/Puerto Rico/8/34(H1N1) (PR8) virus and prophylactically treated with oseltamivir phosphate. (XLSX 287 kb).
ESM_4.xlsx Selected candidate miRNA expression profile comparison of Balb/c mice infected with 1000 TCID50 of mouse adapted A/Puerto Rico/8/34(H1N1) (PR8) virus and therapeutically treated with oseltamivir phosphate. (XLSX 307 kb).
ESM_5.xlsx Selected candidate miRNA expression profile comparison of Balb/c mice infected with 100 TCID50 of mouse adapted A/Puerto Rico/8/34(H1N1) (PR8) virus and therapeutically treated with oseltamivir phosphate. (XLSX 379 kb).

## Figures and Tables

**Table 1:** Significantly dysregulated circulating miRNAs in oseltamivir treated/untreated mice infected with lethal influenza A virus.

	Prophylactic group (1000 TCID50)	Therapeutic group (1000 TCID50)	Therapeutic group (100 TCID50)
Day 1	**miR-150**, **miR-20a**, **miR-221**, **miR-223**, **miR-342-3p**, **miR-423-3p**, **miR-423-5p**, **miR-484**, **miR-486**, **miR-574-3p**, miR-1896, **miR-1897-5p**, **miR-202**, **miR-877**	NS	miR-484

Day 2	miR-1896, miR-345	NS	NS

Day 3	miR-351, miR-503, miR-877	miR-130a, miR-150, miR-199a-3p, miR-22, miR-23a, miR-23b, miR-342-3p, miR-365, miR-382, miR-423-3p, miR-574-3p, miR-345, miR-345-5p, and miR-34c	NS

Day 4	miR-150, miR-23a, miR-351	miR-150, miR-23a, miR-342-3p, miR-382, miR-423-3p, miR-433, miR-484, miR-486, miR-1896, miR-345, miR-34c, miR-503, miR-685, and miR-877	miR-423-3p, miR-433, **miR-574-3p**, and miR-877

Day 5	let-7e, miR-150, miR-22, miR-23a, miR-365, miR-574-3p, miR-1896, miR-345, miR-34c, miR-503	NS	let-7e, miR-130a, miR-150, miR-200b, miR-20a, miR-22, miR-221, miR-223, miR-23a, miR-23b, miR-382, miR-433, miR-486, miR-1897-5p, miR-202, miR-503, and miR-877

Day 6	let-7e, miR-200b, miR-20a, miR-22, miR-221, miR-1896, **miR-685**	miR-199a-3p, miR-342-3p, miR-365, miR-345-5p, miR-34c, and miR-706	miR-223, miR-23b, and miR-1897-5p

Day 7	—	miR-130a, miR-199a-3p, miR-200b, miR-20a, miR-22, miR-221, miR-223, miR-23b, miR-342-3p, miR-365, miR-382, and miR-345	miR-365

Day 8	—	—	miR-351

Day 9	—	—	**miR-130a**, **miR-223**, miR-23b, **miR-342-3p**, miR-382, **miR-486**, miR-1897-5p, **miR-345-5p**, and **miR-877**

Statistically significant unique miRNA expression patterns (*p* < 0.05 and 0.01) from days 1 to 9 in the plasma of mice infected with lethal mouse adapted influenza A/Puerto Rico/8/34 (H1N1) virus and treated with oseltamivir phosphate either prophylactically or therapeutically compared with the corresponding plasma miRNAs of infected mice. For the prophylactic group, mice were infected with 1000 TCID50 virus and treated 2 h before infection with oseltamivir. The therapeutic groups were infected with 1000 or 100 TCID50 virus and treated 24 h postinfection. Prophylactic and therapeutic groups were administered with oseltamivir 10 mg/kg by oral gavage twice daily for 5 days. An infected group gavaged with distilled water was added as control. The miRNAs shown in bold were downregulated. NS: no significant miRNAs detected.
