# Supplementary material for: The Effect of Oseltamivir on the Disease Progression of Lethal Influenza A Virus Infection: Plasma Cytokine and miRNA Responses in a Mouse Model
Source: Dis Markers. 2016 Mar 8;2016:9296457. doi: 10.1155/2016/9296457 (PMC4824134; doi:10.1155/2016/9296457)
Supplement: Supplementary file 1 — ESM_1.docx Effect of therapeutic oseltamivir treatment on cytokine levels in lethal influenza A virus infected mice. Changes in cytokine concentrations [TNF-α (a), IL-1β (b), and IL-12p70 (c)] over time in plasma samples of mice infected with 100 TCID50 lethal mouse adapted influenza A/Puerto Rico/8/34 (H1N1) virus and treated therapeutically either with oseltamivir phosphate or water. Therapeutic groups (Δ) were administered 24 h post-infection with oseltamivir 10 mg/kg by oral gavage twice daily for 5 days. An infected group (■) gavaged with distilled water was added as control for each experiment. On day 1 – 10 post-infection, plasma samples were collected from sacrificed mice and cytokine levels were measured by Mesoscale ELISA. Levels were indicated as pg/ml of plasma. For the purpose of analysis, cytokine levels below the detection limit were set to the lower limit of detection in each case. (DOCX 164 kb). ESM_2.xlsx Global rodent miRNA expression profile of healthy and A/Puerto Rico/8/34(H1N1) (PR8) virus (100 and 1000 TCID50) infected Balb/c mouse plasma samples. (XLSX 135 kb). ESM_3.xlsx Selected candidate miRNA expression profile comparison of Balb/c mice infected with 1000 TCID50 of mouse adapted A/Puerto Rico/8/34(H1N1) (PR8) virus and prophylactically treated with oseltamivir phosphate. (XLSX 287 kb). ESM_4.xlsx Selected candidate miRNA expression profile comparison of Balb/c mice infected with 1000 TCID50 of mouse adapted A/Puerto Rico/8/34(H1N1) (PR8) virus and therapeutically treated with oseltamivir phosphate. (XLSX 307 kb). ESM_5.xlsx Selected candidate miRNA expression profile comparison of Balb/c mice infected with 100 TCID50 of mouse adapted A/Puerto Rico/8/34(H1N1) (PR8) virus and therapeutically treated with oseltamivir phosphate. (XLSX 379 kb). [file 9296457.f1.zip › 9296457.f1/ESM_1.docx]

**The effect of oseltamivir on the disease progression of lethal influenza A virus infection: Plasma cytokine and miRNA responses in a mouse model**

Ashok K Chockalingam^1^, Salaheldin Hamed^2^, David G Goodwin^5^, Barry A Rosenzweig^1^, Eric Pang^3^, Michael T Boyne 2nd^4#^ and Vikram Patel^1^*

Author affiliations

^1^DARS/OCP/OTS/CDER, ^2^OCP/OTS/CDER, ^3^OPQ/OLDP/CDER, ^4^DPA/OTR/OPS/CDER, ^5^DMD/OIR/CDRH, US Food and Drug Administration, Silver Spring, Maryland 20993 USA.

# Present address: BioTechLogic, 717, Indian road, Glenview, IL 60025 USA.

*E-mail address for corresponding author: [Vikram.Patel@fda.hhs.gov](mailto:Vikram.Patel@fda.hhs.gov)

**Fig. ESM_1** Effect of therapeutic oseltamivir treatment on cytokine levels in lethal influenza A virus infected mice


Effect of therapeutic oseltamivir treatment on cytokine levels in lethal influenza A virus infected mice. Changes in cytokine concentrations [TNF-α (a), IL-1β (b), and IL-12p70 (c)] over time in plasma samples of mice infected with 100 TCID50 lethal mouse adapted influenza A/Puerto Rico/8/34 (H1N1) virus and treated therapeutically either with oseltamivir phosphate or water. Therapeutic groups (Δ) were administered 24 h post-infection with oseltamivir 10 mg/kg by oral gavage twice daily for 5 days. An infected group (◼) gavaged with distilled water was added as control for each experiment. On day 1 – 10 post-infection, plasma samples were collected from sacrificed mice and cytokine levels were measured by Mesoscale ELISA. Levels were indicated as pg/ml of plasma. For the purpose of analysis, cytokine levels below the detection limit were set to the lower limit of detection in each case.

**C**

**B**

**A**
